# Monoclonal Antibody Treatments for Alzheimer's Disease: Aducanumab and Lecanemab

**DOI:** 10.15190/d.2023.12

**Published:** 2023-09-25

**Authors:** Selia Chowdhury

**Affiliations:** ^*^Dhaka Medical College, Dhaka, Bangladesh

**Keywords:** Alzheimer's disease, dementia, aducanumab, lecanemab, amyloid-beta antagonist.

## Abstract

Alzheimer's disease (AD) has witnessed a gradual rise in its prevalence in recent decades, particularly impacting a substantial segment of individuals aged 85 and above. The core pathological features of AD involve the presence of amyloid-β (Aβ) plaques and neurofibrillary tangles formed due to the hyperphosphorylation of tau protein. Current AD treatments primarily provide symptomatic relief without addressing the fundamental disease progression. Given the sluggish pace of finding a definitive AD cure, research has shifted its focus towards pioneering approaches. There is an increasing emphasis on targeting the early stages of AD, with the aim of intervening before irreversible pathological changes take hold, thus preserving cognitive function and neuronal health. In recent years, significant strides have been made in the development and subsequent clinical testing of disease-modifying therapies (DMTs) designed to potentially alter the underlying pathophysiology of AD. These therapeutic strategies involve the utilization of monoclonal antibodies (mAbs) specifically directed at Aβ. Some of the drugs falling into this category include aducanumab, bapineuzumab, gantenerumab, solanezumab, and lecanemab. These treatment approaches are grounded in the hypothesis that a systemic failure in clearing Aβ contributes to the initiation and progression of AD. Recently, aducanumab and lecanemab have received FDA approval for the treatment of AD with mild cognitive impairment. This review offers a comprehensive summation of recent research endeavors that delve into the therapeutic effects and clinical trial outcomes of aducanumab and lecanemab in individuals afflicted by AD.

## SUMMARY

1. Introduction

2. Brief overview of aducanumab and lecanemab

3. Phase 3 Randomized Control Trials

4. Efficacy

5. Safety

6. Conclusion

## 1. Introduction

Alzheimer's disease (AD) is a progressive and irreversible neurodegenerative condition, representing the primary age-related disease^1^ and responsible for about 64% of dementia cases^2^. In individuals with Alzheimer’s, there's a transformation of the regular soluble amyloid β peptide into toxic oligomeric amyloid β or into fibrillar amyloid β, leading to the formation of amyloid plaques and congophilic angiopathy^3^. Additionally, abnormally phosphorylated tau accumulates in the form of toxic oligomers and neurofibrillary tangles^3^. The key driver of Alzheimer's pathogenesis is suggested to be the accumulation of amyloid β, which occurs as a result of an uneven equilibrium between its production and elimination in the brain^4^. Preclinical studies have shown that anti-amyloid β antibodies can inhibit amyloid β peptide fibril formation, break down pre-formed fibrils, and prevent neurotoxicity in cell culture^3,5^.

Recently, the food and drug administration (FDA) has approved two amyloid β-directed antibodies, aducanumab and Lecanemab, for early Alzheimer's treatment, bringing hope after a long period without new approvals. On June 7, 2021^6^, aducanumab received its initial FDA approval for Alzheimer's treatment in the USA. Aducanumab is a human monoclonal antibody of the IgG1 class targeting aggregated forms of amyloid β, both soluble and insoluble^7^. In Alzheimer's patients, aducanumab reduces brain amyloid β levels in a dose- and time-dependent manner^8^.

Very recently, on January 6, 2023, the U.S. FDA granted approval for early Alzheimer’s treatment under the Accelerated Approval pathway^9^. Lecanemab is a humanized IgG1 version of the murine antibody mAb158, primarily focused on soluble Aβ protofibrils while still effective against insoluble fibrils. Aβ protofibrils are sizable, soluble Aβ aggregates that induce neurotoxicity by interfering with memory-related electrophysiological systems^10^. Lecanemab has demonstrated its ability to reduce pathogenic Aβ, prevent Aβ deposition, and specifically reduce Aβ protofibrils in the brains and cerebrospinal fluid of animal models with AD^11^. The specific targeting of Aβ protofibrils distinguishes lecanemab from other anti-amyloid monoclonal antibodies.

Consequently, the primary objective of this review is to conduct a thorough and in-depth assessment of the current body of evidence pertaining to the utilization of lecanemab and aducanumab for the treatment of AD. This review seeks to examine all available research, clinical trials, and studies to provide a comprehensive understanding of the efficacy, safety, and overall impact of these two treatments in addressing the complexities of AD.

## 2. Brief overview of aducanumab and lecanemab

Extensive evidence supports the hypothesis that Aβ plays a causative and initiating role in the development of AD. Nevertheless, only a limited number of anti-amyloid agents have demonstrated significant effectiveness in clinical trials^12^. Aducanumab and lecanemab are monoclonal antibodies, either human or humanized, that exhibit strong binding to aggregated Aβ, facilitating its removal through Fc receptor-mediated phagocytosis. Importantly, these antibodies exhibit notably lower affinity for Aβ monomers^8,12,13,14,15^. However, they differ in their preference for targeting soluble Aβ oligomers as opposed to plaques or fibrils. In a direct comparative study, lecanemab exhibited a tenfold higher selectivity for oligomers when compared to aducanumab^8,13,14^.

Preclinical investigation conducted in a transgenic mouse model of AD demonstrated that aducanumab could penetrate the brain, binding to parenchymal amyloid β and effectively reducing both soluble and insoluble amyloid β in a time- and dose-dependent manner^16^. Another study revealed that chronic systemic administration of aducanumab in a transgenic mouse model inhibited Aβ toxicity, enhancing phagocytosis and cell viability in the vicinity of senile plaques, indicating a potential positive impact on the proteome of these plaques and their surrounding tissue^15^. Additionally, aducanumab was found to restore disrupted calcium homeostasis caused by amyloid β in a transgenic AD model^17^.

In a transgenic mouse model characterized by Aβ overexpression, the murine counterpart of lecanemab, mAb158, administered via intraperitoneal injection, exhibited a remarkable affinity for protofibrils over monomers, resulting in a dose-dependent reduction in brain protofibril levels^18^. The favorable outcomes of these medications observed in preclinical studies prompted the progression to clinical trials.

Both medications are administered through IV infusion therapy for individuals with mild cognitive impairment (MCI) or mild dementia due to AD, a distinction from the indications of certain standard of care treatments. There is currently no available data regarding the safety and efficacy of initiating treatment at earlier or later stages of disease progression^19^. The dosing regimen for lecanemab is 10 mg/kg administered once every 2 weeks, while aducanumab allows for a titration regimen, with the possibility of up to seven infusions to achieve the target dose of 10 mg/kg IV every 4 weeks^19^. A detailed comparison between the two drugs is provided in the Table 1.

**Table 1:** Comparison of key characteristics ARIA: Amyloid-related imaging abnormalities. *Time to peak brain steady state concentration (without titration) = 5 times plasma t_1/2_ or 5 times dosing frequency, if dosing interval is longer than plasma t_1/2_ Data sources: Reference^12, 19^.

	Aducanumab	Lecanemab
Type of antibody	IgG1 human N-terminal	IgG1 humanized N-terminal
Target amyloid species	Plaque, fibrils > > oligomers	Large oligomers (protofibrils) > plaque
Dose	Titrate up to 10 mg/kg	10 mg/kg
Dose frequency	Once every 4 weeks	Once every 2 weeks
Route	Intravenous	Intravenous
Titration	Over 6 months	No titration
Plasma half-life	21 days	5.3 days
Brain penetration	< 1.5%	~ 0.5%
Time to peak brain steady state exposure*	~ 5 months	~ 2.5 months
Most common adverse effects	ARIA Headache	ARIA Headache Infusion related reactions

## 3. Phase 3 Randomized Control Trials

Two identically designed randomized, double-blind, placebo-controlled, multicenter studies, EMERGE (NCT02484547) and ENGAGE (NCT02477800), investigated the efficacy of intravenous aducanumab in patients with early AD^20^. These research studies were conducted at 348 different locations across 20 countries. The participants, totaling 1,638 in the EMERGE study and 1,647 in the ENGAGE study, were individuals aged 50 to 85 years who had confirmed amyloid pathology and met the clinical criteria for either mild cognitive impairment due to AD or mild AD dementia. A detailed caracteristics of the patients are described in Table 2. Of these participants, 55.2% (1,812 individuals) successfully completed the entire study. The participants were randomly assigned to one of three groups in a 1:1:1 ratio: receiving aducanumab at a low dose (with a target dose of 3 or 6 mg/kg), aducanumab at a high dose (with a target dose of 10 mg/kg), or a placebo. These treatments were administered through intravenous infusion once every 4 weeks over a period of 76 weeks. The primary endpoint was the change from baseline in the Clinical Dementia Rating-Sum of Boxes (CDR-SB) at week 78. A subset of patients underwent amyloid positron emission tomography (PET) imaging to assess the impact of aducanumab on amyloid β plaque levels in the brain. The PET signal was quantified using the SUVR method, and the results were expressed on the Centiloid scale. Both studies were halted in March 2019 based on a prespecified interim analysis for futility.

**Table 2:** Description of Trials and Characteristics of Trial Participants MMSE: Mini-Mental State Examination, CDR-SB: Clinical Dementia Rating-Sum of Boxes, SD: Standard deviation. * MMSE: A 30-item screening tool categorizes cognitive impairment as severe (≤9 points), moderate (10–20 points), mild (21–24 points), and normal cognition (25 points and above). ** CDR-SB scores of 0, 0.5, 1.0–2.5, 2.5–4.0, and ≥4.5 were categorized as normal cognition, subjective cognitive decline, mild cognitive impairment, very mild dementia, and dementia, respectively.

Trial	Phase	Dose	No of Participants, n	Female, n (%)	Age (mean±SD, years)	White, n (%)	MMSE* (mean±SD)	CDR-SB score** (mean±SD)
Clarity AD	III	10 mg/kg (biweekly)	859	443 (51.6)	71.4±7.9	655 (76.3)	25.5 (SD: 2.2)	3.17±1.34
		Placebo	875	464 (53.0)	71.0±7.8	677 (77.4)	25.(SD: 2.2)	3.22±1.34
EMERGE	III	Placebo	548	290 (53)	70.8±7.4	431 (79)	26.4±1.8	2.47±1.00
		Low dose	543	269 (50)	70.6±7.4	432 (80)	26.3±1.7	2.46±1.01
		High dose	547	284 (52)	70.6±7.5	422 (77)	26.3±1.7	2.51±1.05
ENGAGE	III	Placebo	545	287 (53)	69.8±7.7	413 (76)	26.4±1.7	2.40±1.01
		Low dose	547	284 (52)	70.4±7.0	412 (75)	26.4±1.8	2.43±1.01
		High dose	555	292 (53)	70.0±7.7	413 (74)	26.4±1.8	2.40±1.01

**Table 3:** Key characteristics of randomized clinical trials of lecanemab AMC: Adjusted mean change, AMD: Adjusted mean difference, CDR-SB: Clinical Dementia Rating-Sum of Boxes, TRAEs: Treatment-related adverse events, SAEs: Serious adverse events.

Study	Dose	CDR-SB score		Incidence of TRAEs, n (%)	ARIA-E, n (%)	Microhemorrhage, n (%)	Incidence of treatment-related SAEs, n (%)
Clarity AD	Placebo (AMC)	1.66	At 18 months	197 (22.0)	15 (1.7)	69 (7.7)	101 (11.3)
	10 mg/kg (AMD vs. placebo (95% CI))	−0.45 (−0.67 to −0.23)		401 (44.7)	113 (12.6)	126 (14.0)	126 (14.0)
EMERGE	Placebo (decline±; SE)	1.74±;0.11	At 78 weeks	-	13 (2)	37 (7)	81 (15)
	Low dose (Difference vs placebo (%))	-0.26 (-15%)		-	140 (26)	87 (16)	72 (13)
	High dose (Difference vs placebo (%))	-0.39 (-22%)		-	188 (35)	108 (20)	73 (13)
ENGAGE	Placebo (decline±; SE)	1.56±;0.11		-	16 (3)	34 (6)	70 (13)
	Low dose (Difference vs placebo (%))	-0.18 (-12%)		-	141 (26)	89 (16)	76 (14)
	High dose (Difference vs placebo (%))	0.03 (2%)		-	199 (36)	104 (19)	79 (14)

The clinical trial for lecanemab (Table 3), known as Clarity AD (NCT03887455), enrolled 5967 individuals who underwent screening, and out of them, 1795 were randomized^21^. The participants, aged between 50 to 90 years, had early AD, characterized by mild dementia or mild cognitive impairment due to AD, with evidence of amyloid presence confirmed through PET or cerebrospinal fluid (CSF) testing. Among the 1795 participants, 898 were randomly assigned to receive lecanemab (10 mg/kg of body weight every 2 weeks), while 897 received a placebo. The enrollment took place at 235 sites in North America, Europe, and Asia from March 2019 to March 2021. Ultimately, 729 participants in the lecanemab group and 757 in the placebo group completed the trial and provided data on the primary endpoint.

## 4. Efficacy

The primary endpoint of EMERGE and ENGAGE was the change from baseline in the CDR-SB at week 78. In the EMERGE study, the primary endpoint was successfully achieved, with high-dose aducanumab demonstrating a significant reduction (a 22% decrease) in comparison to placebo (a difference of -0.39; 95% CI, -0.69 to -0.09; P=.012)). Low-dose aducanumab did not differ significantly from placebo. Amyloid PET scans revealed a dose- and time-dependent reduction in Aβ pathology with aducanumab, and CSF biomarkers also improved in both high and low-dose groups.

In contrast, the ENGAGE study did not meet its primary endpoint, with neither high nor low-dose aducanumab showing a significant difference in CDR-SB scores compared to placebo (a difference of 0.03; 95% CI, -0.26 to 0.33; P=.833; 2% increase). However, a subgroup receiving at least 14 doses of high-dose aducanumab did show improvement, similar to the EMERGE study. Amyloid PET scans also showed a reduction in amyloid β pathology, but CSF biomarkers did not significantly change.

The primary endpoint of the Clarity AD study was the change in the CDR-SB score at 18 months.

The adjusted mean change from baseline at 18 months in the CDR-SB score was 1.21 in the lecanemab group and 1.66 in the placebo group, showing a statistically significant difference of -0.45 (95% confidence interval [CI], -0.67 to -0.23; P<0.001) in favor of lecanemab. The study also examined several secondary endpoints, including the change in amyloid burden on PET, the Alzheimer's Disease Assessment Scale-Cognitive Subscale 14 (ADAS-cog14), the Alzheimer's Disease Composite Score (ADCOMS), and the Alzheimer's Disease Cooperative Study-Mild Cognitive Impairment Activities of Daily Living (ADCS-MCI-ADL) score, all of which demonstrated significantly better outcomes in the lecanemab group compared to the placebo group.

## 5. Safety

In terms of safety, intravenous aducanumab at a dose of 10 mg/kg was generally well tolerated, with some common adverse reactions. The most frequently reported adverse reactions in aducanumab recipients, with an incidence of 2% or higher than in the placebo group, encompassed amyloid-related imaging abnormalities-oedema (ARIA-E; 35% vs. 3%), headache (21% vs. 16%), amyloid-related imaging abnormalities hemosiderin deposition (ARIA-H) microhemorrhage (19% vs. 7%), ARIA-H superficial siderosis (15% vs. 2%), falls (15% vs. 12%), diarrhea (9% vs. 7%), and confusion/delirium/altered mental status/ disorientation (8% vs. 4%). Adverse reactions led to treatment discontinuation in some patients, with ARIA-H superficial siderosis being the most common reason for discontinuation.

In the Clarity AD study, adverse events were observed in 88.9% of the 898 recipients who received lecanemab at a dose of 10 mg/kg every 2 weeks, while 81.9% of the 897 individuals who received a placebo experienced adverse events. Among the adverse events reported, those with an incidence of more than 10% in the lecanemab 10 mg/kg every 2 weeks group included infusion-related reactions (IRRs) (26.4% compared to 7.4% in the placebo group), amyloid-related imaging abnormalities with cerebral microhemorrhages, cerebral macrohemorrhages, or ARIA-H (17.3% compared to 9.0%), amyloid-related imaging abnormalities with edema (ARIA-E) (12.6% compared to 1.7%), headache (11.1% compared to 8.1%), and falls (10.4% compared to 9.6%). Infusion-related reactions were mostly mild to moderate, and preventative medications were not used by over half of the participants who did not experience any reactions after the first dose. The incidence of ARIA-E and ARIA-H tended to be higher among ApoE ε4 carriers than noncarriers, and higher among ApoE ε4 homozygotes than heterozygotes.

The EMERGE, ENGAGE, and Clarity AD studies have limitations, including relatively short treatment durations (up to 18 months), challenges due to the Covid-19 pandemic, high dropout rates (17.2%), and small sample sizes in certain biomarker substudies. These trials also lack diversity in their study populations, particularly in terms of racial/ethnic representation and comorbid conditions, raising concerns about generalizability. Further data collection is needed to address these limitations and provide more comprehensive insights.

## 6. Conclusion

In recent years, a new class of highly specific mAbs targeting Aβ has emerged as potential DMTs for AD. Notably, among these mAbs, aducanumab and lecanemab have demonstrated relatively promising effects. However, the effectiveness of these mAbs in AD patients remains uncertain, and several questions remain unanswered. While aducanumab boasts higher brain penetration compared to lecanemab, this advantage does not necessarily translate into superior efficacy, underscoring the importance of lecanemab's enhanced selectivity for Aβ oligomers^12^. Furthermore, lecanemab's dosing regimens in both phase 2 and 3 trials are approximately double that of aducanumab, which may contribute to some of the observed differences in efficacy^12^.

Overall, aducanumab marks the first approved mAb for DMTs in mild AD. The outcomes of the twin phase III studies for aducanumab (EMERGE and ENGAGE) present a somewhat ambiguous but encouraging picture. While one trial yielded positive results (EMERGE), the other (ENGAGE) was decidedly negative with regard to the primary clinical endpoint, which measured the slowing of cognitive decline using the CDR scale. These results fall short of the typical standards for marketing authorization^22^. Nevertheless, subgroup analyses and investigations of tau pathology biomarkers suggest the emergence of a potential clinical effect^22^. To address the ambiguity in the outcomes of EMERGE and ENGAGE, the company plans to initiate a new large-scale global phase III randomized-controlled trial, with results anticipated in 2026.

Lecanemab emerges as a promising AD treatment among these mAbs due to its ability to reduce brain Aβ levels, alleviate cognitive decline, and exhibit a lower incidence of ARIA-E. It demonstrates a moderate therapeutic effect with enhanced safety. However, results from several clinical trials predominantly yielded negative outcomes, failing to demonstrate clinically relevant effects in patients with clinically evident or prodromal dementia. Further investigations into the efficacy and safety of these mAbs, particularly in asymptomatic Aβ-positive individuals, are warranted. Regarding other molecules, results from phase III trials for donanemab are not expected until 2024. Consequently, envisioning the availability of a disease-modifying treatment for AD in clinical practice in the near future no longer seems unreasonable.

## KEY POINTS - Recommendations

- Aducanumab and lecanemab are monoclonal antibodies targeting amyloid-β (Aβ) with the potential to modify the progression of AD

- Aducanumab demonstrated in phase III trials (EMERGE) a reduction in cognitive decline, but the other trial (ENGAGE) did not meet its primary endpoint, raising questions about its overall effectiveness

- Lecanemab, with its focus on Aβ protofibrils, shows promise in reducing Aβ levels, cognitive decline, and safety compared to aducanumab, but clinical trials have yielded mixed results, especially in patients with advanced AD
